# A novel dataset of annotated oyster mushroom images with environmental context for machine learning applications

**DOI:** 10.1016/j.dib.2024.111074

**Published:** 2024-10-28

**Authors:** Sonay Duman, Abdullah Elewi, Abdulsalam Hajhamed, Rasheed Khankan, Amina Souag, Asma Ahmed

**Affiliations:** a Computer Engineering Department, Mersin University, 33343 Mersin, Turkey; b Faculty of Agricultural Sciences, University of Hohenheim, 70599 Stuttgart, Germany; c Software Engineering Department, Toros University, 33140 Mersin, Turkey; d Electrical and Electronic Engineering Department, Mersin University, 33343 Mersin, Turkey; e School of Engineering, Technology and Design, Canterbury Christ Church University, Canterbury, United Kingdom; f Department of Chemical and Environmental Engineering, University of Nottingham, Nottingham NG7 2RD, United Kingdom

**Keywords:** Oyster mushroom, Mushroom maturity, Smart farming, Precision agriculture, Image classification, Feature extraction, YOLO, PASCAL VOC

## Abstract

State-of-the-art technologies such as computer vision and machine learning, are revolutionizing the smart mushroom industry by addressing diverse challenges in yield prediction, growth analysis, mushroom classification, disease and deformation detection, and digital twinning. However, mushrooms have long presented a challenge to automated systems due to their varied sizes, shapes, and surface characteristics, limiting the effectiveness of technologies aimed at mushroom classification and growth analysis. Clean and well-labelled datasets are therefore a cornerstone for developing efficient machine-learning models. Bridging this gap in oyster mushroom cultivation, we present a novel dataset comprising 555 high-quality camera raw images, from which approximately 16.000 manually annotated images were extracted. These images capture mushrooms in various shapes, maturity stages, and conditions, photographed in a greenhouse using two cameras for comprehensive coverage. Alongside the images, we recorded key environmental parameters within the mushroom greenhouse, such as temperature, relative humidity, moisture, and air quality, for a holistic analysis. This dataset is unique in providing both visual and environmental time-point data, organized into four storage folders: “Raw Images”; “Mushroom Labelled Images and Annotation Files”; “Maturity Labelled Images and Annotation Files”; and “Sensor Data”, which includes time-stamped sensor readings in Excel files. This dataset can enable researchers to develop high-quality prediction and classification machine learning models for the intelligent cultivation of oyster mushrooms. Beyond mushroom cultivation, this dataset also has the potential to be utilized in the fields of computer vision, artificial intelligence, robotics, precision agriculture, and fungal studies in general*.*

Specifications Table

**Table:** 

Subject	Agriculture Engineering, Computer Vision, Artificial Intelligence
Specific subject area	Machine learning-based mushroom detection and classification
Type of data	Image, Excel File, Annotation File
Data collection	The raw images were captured in an oyster mushroom farm using two TP-Link Tapo C310 IP cameras, each set to full HD resolution of 1920 × 1080, resulting in a minimum of 4–5 images per day during two cultivation cycles from late December 2022 until late April 2023. Environmental parameters were recorded concurrently using various sensors. These include temperature, relative humidity, and air quality inside the greenhouse, in addition to temperature and moisture inside mushroom composite bags. The images and sensor readings were time-stamped to be linked together. Mushroom images were manually extracted from raw images, maturity-labelled and annotated using different annotation formats*.*
Data source location	City/Region: Mersin/Akdeniz Country: Turkey Latitude and longitude for collected samples/data: *36°49′49.8″N 34°43′20.4″E*
Data accessibility	Repository name: Mendeley Data Data identification number: 10.17632/hf55tkx489.1 Direct URL to data: https://data.mendeley.com/datasets/hf55tkx489/1 Instructions for accessing these data:
Related research article	None*.*

## 1 Value of the Data

• The diverse features of the mushrooms in this dataset are crucial for any intelligent system, as they offer comprehensive information to cover a wide range of mushroom sizes, varied shapes, and growth stages.
• This data can be valuable for researchers in the fields of computer vision, agricultural robotics, mushroom classification, and fungal studies.
• Farmers and agriculturalists engaged in smart farming and precision agriculture, especially those working within greenhouses, can harness this data to support their specific needs and research endeavors.
• About 16,000 oyster mushroom images, labelled as “mushroom”, “mature” and “immature”, from different shapes, sizes, day times and maturity stages, were manually extracted and annotated to help researchers in mushroom classification and growth analysis problems.
• The dataset of mushroom images was captured from the day of mushroom appearance through to harvesting, along with the corresponding environmental parameters within the mushroom greenhouse. This will help researchers identify correlation patterns between the mushrooms and their surrounding environmental conditions.
• The dataset includes annotated mushroom images, providing a valuable resource for developing and refining machine learning models for classification and regression applications.

## 2 Background

Automated systems often struggle with the classification and growth analysis of mushrooms, primarily due to the wide range of sizes, shapes and surface characteristics they exhibit. This inherent diversity has posed a significant challenge for mushroom-related technologies. Recent research [1] has shown that machine and deep learning technologies hold great promise in overcoming this challenge, given their high precision and accuracy. However, one of the key limitations has been the lack of diverse and comprehensive image datasets that include labelled mushrooms across various stages, shapes, and sizes, which are essential for training and validating machine learning models. To bridge this gap, we have curated a dataset that includes high-quality images of meticulously annotated mushrooms, capturing different shapes, maturity stages, and conditions. Additionally, we have matched these images with corresponding environmental parameters from the farm, ensuring thorough and extensive coverage of the topic.

## 3 Data Description

**Fig. 1:**
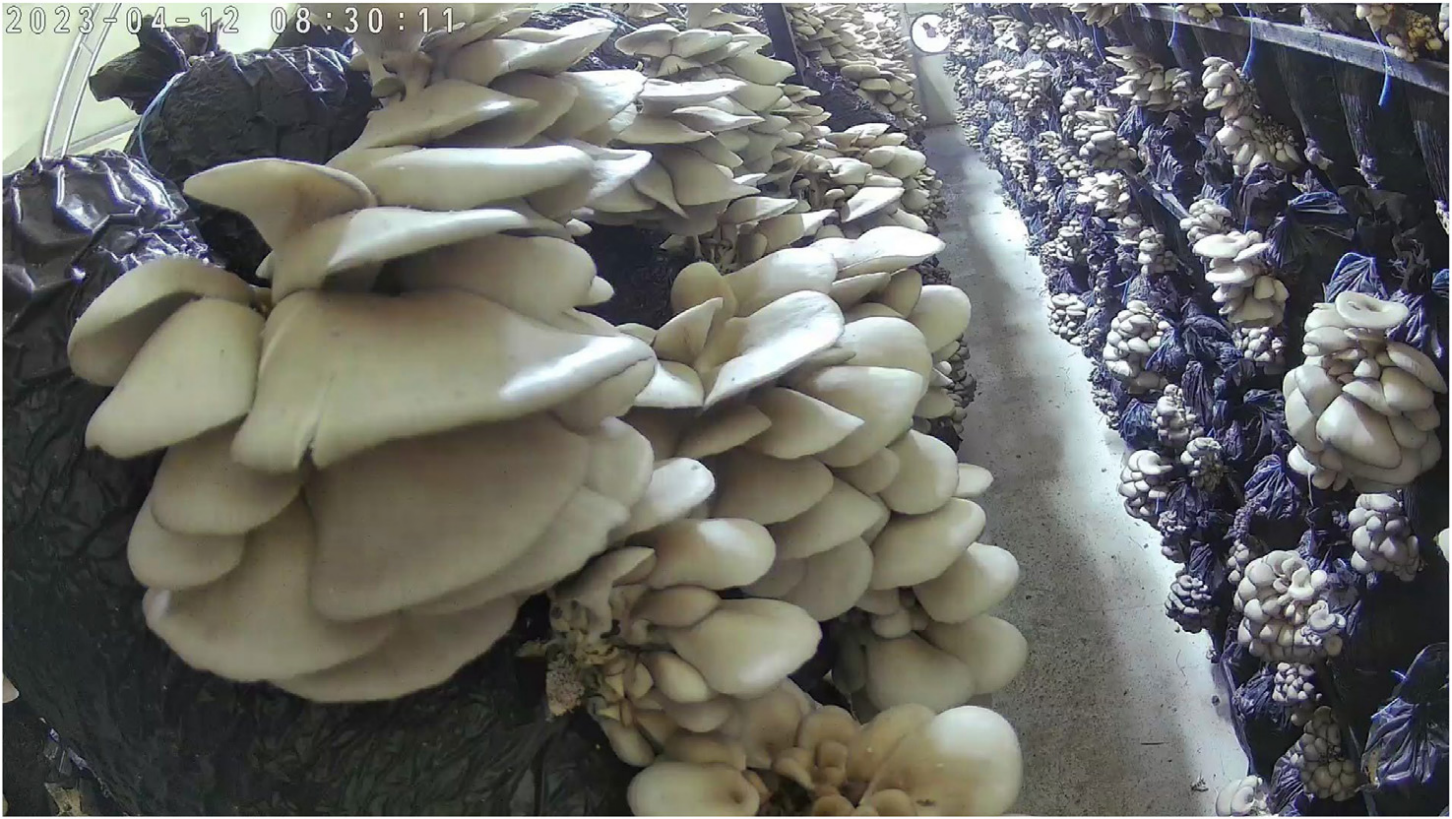
An example of original camera images.

**Fig. 2:**
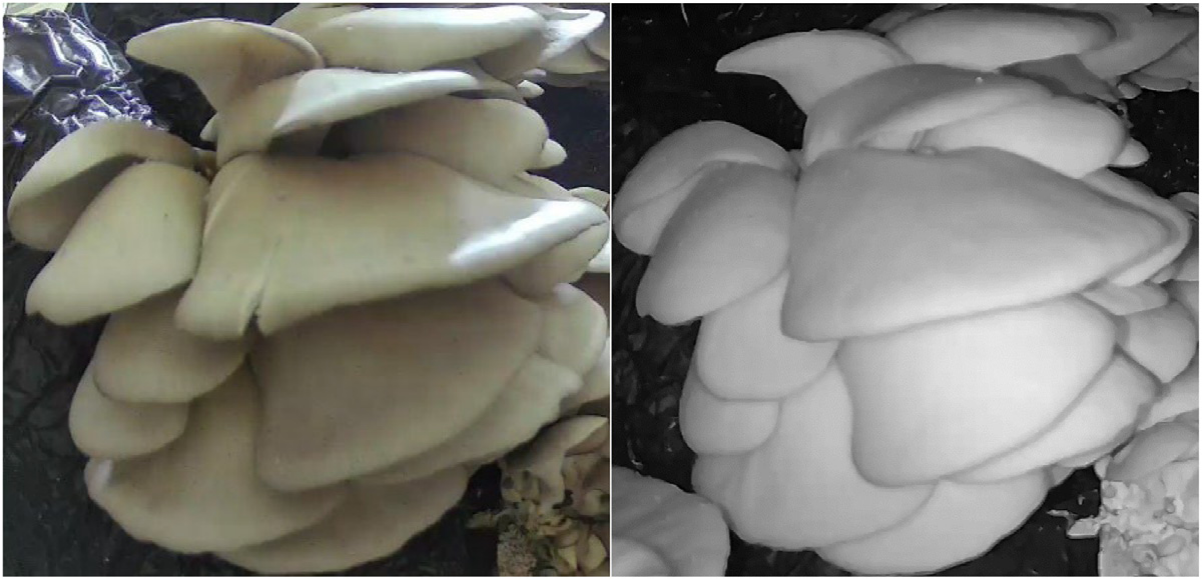
An example of manually extracted images of the same mushroom during daytime (left) and at night (right).

The meticulously gathered dataset has been created with the aim of addressing the existing challenges in mushroom analysis systems. These challenges revolve around the various states of mushrooms, including their growth, maturity, surface conditions, and other factors that can hinder the accuracy of classification systems. Fig. 1 shows an example of raw camera images, and Fig. 2 shows mushroom images that were extracted from the raw camera images.

The oyster mushroom dataset comprises 555 raw camera images, approximately 8000 mushroom-labelled images, about 8000 maturity-labelled images, various annotation files, and sensor data. The images were captured in both day and night-time settings in an oyster mushroom cultivation greenhouse/farm in Mersin, on Turkeyʼs Mediterranean coast. A remote monitoring and management (RMM) system was designed and implemented in the farm for two cultivation cycles from late December 2022 until late April 2023. More details about the system are available in our previous work [2]. Different internet of things (IoT) platforms, such as ThingSpeak™ and Arduino IoT Cloud™, were trialled in the first cycle. ThingSpeak platform was the main IoT platform used in the second cycle due to its superior affordances.

All raw camera images and various sensor recording data were time-stamped for correlation and analysis. The “Sensor Data” folder contains two sub-folders for every cultivation cycle. The “Cycle I” sub-folder includes 4 .xlsx files for all the first-cycle ThingSpeak data, and a sub-folder for daily data obtained using Arduino IoT Cloud. The “Cycle II” folder includes 11 .xlsx files of various environmental sensor values, obtained using ThingSpeak from different sensors during the second cycle and presented in separate time-stamped Excel files for easy management and connection with images. For temperature and relative humidity in the greenhouse, DHT21 and SHT20 sensors were utilised. Several DS18B20 sensors were utilised for measuring temperature inside mushroom composite bags, while resistive and capacitive moisture sensors were used for moisture inside bags. For air quality inside the greenhouse, SGP30 and CSS811 sensors were utilised for measuring the estimated CO2 (eCO2) and total volatile organic compounds (tVOC) in the mushroom greenhouse. For managing the sensors, ESP8266 and ESP32 microcontroller boards were utilised. Further technical details can be found in our previous publication [2].

The environmental data from different sensors, in addition to mushroom images, allow researchers to observe how environmental values change instantly and how they affect mushroom growth processes. This comprehensive collection addresses the need for diverse mushroom data, showcasing various stages of maturity, different environmental conditions, and other factors. By encompassing a wide range of conditions and times of day, our dataset equips researchers and practitioners in the field of smart mushroom farming with a valuable resource to enhance the accuracy and effectiveness of their classification and analysis systems.

Within the dataset, numerous mushrooms populate each raw camera image (Fig 2). During the labelling process, the mushrooms that were clearly visible throughout the growth process in the raw images were cut from the images from the first day until the day they were collected and took their place in the annotation file. This ensured that images of each growth period were included in the dataset. The distribution of the maturity labelled images is 3158 “mature” and 5124 “immature” labelled images. As the images were collected frequently from different times of the day, both day images (about 4000) and night images (about 3963) were included in the dataset.

**Fig. 3:**
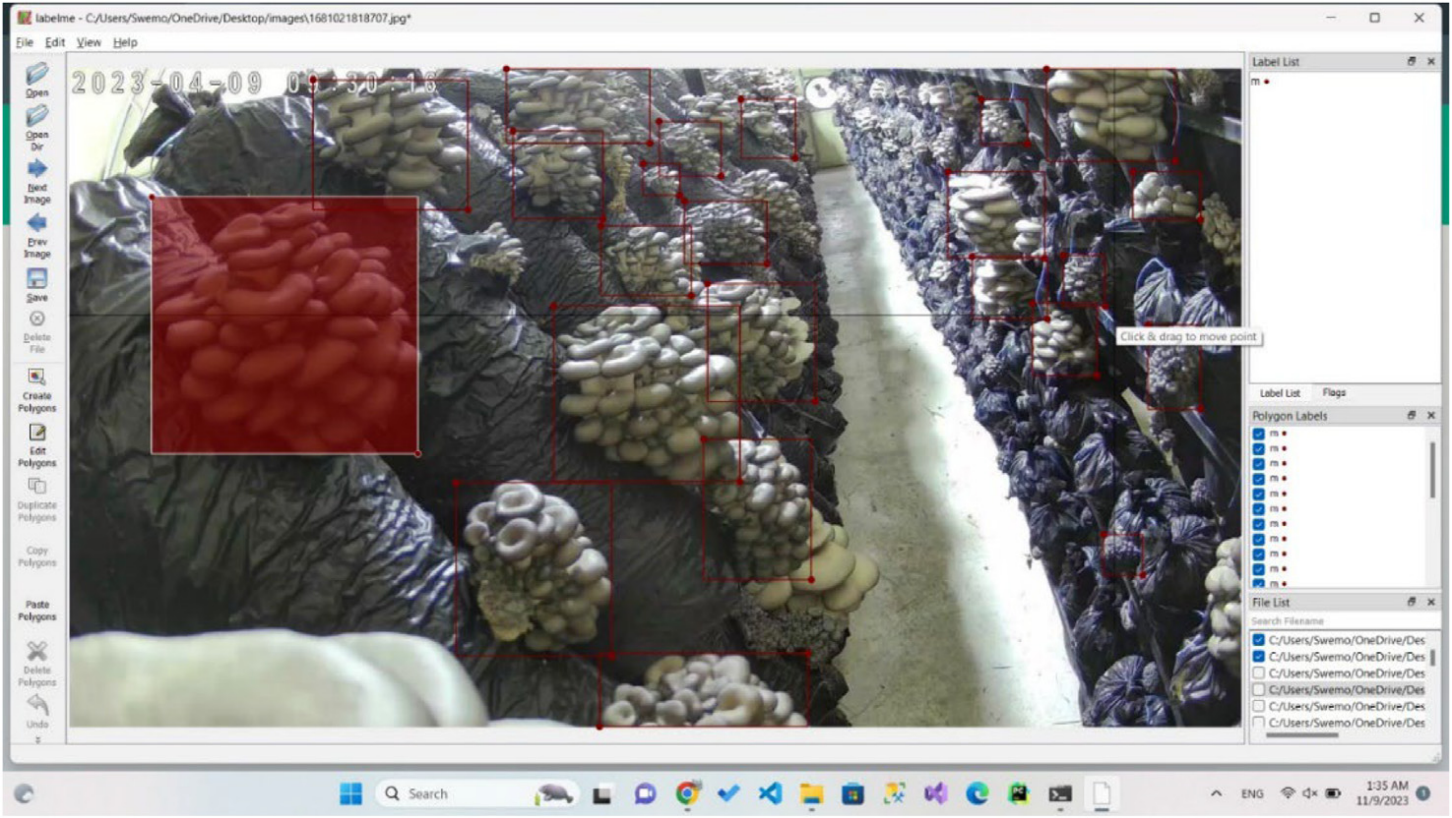
Labelling with LabelMe software.

The images were processed in a manner where they were retained at their original size during the labelling phase. We employed the open-source LabelMe software [3] for this purpose. While labelling the images, we ensured that each bounding box precisely encompassed the entire mushroom, minimizing the inclusion of unnecessary background pixels, as depicted in Fig. 3. The selected annotation formats (JSON, TXT and XML) are widely used across popular object detection environments, providing researchers with an effortless integration of the proposed dataset into their work. This streamlines the training process for object detection models, eliminating the requirement to convert annotation files into various formats.

**Fig. 4:**
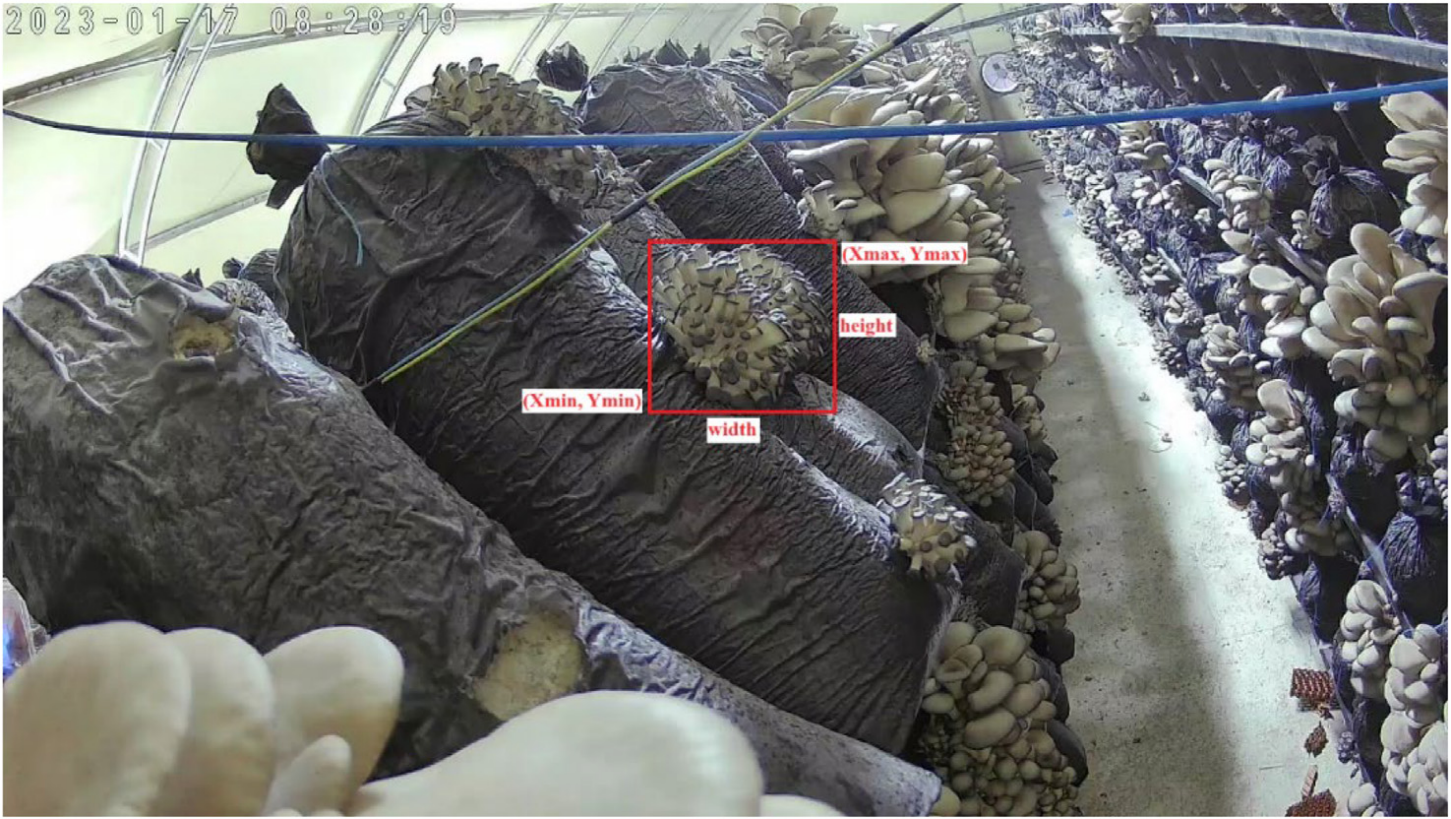
Pascal VOC annotation format.

The Pascal VOC (Visual Object Classes) [4] format is an XML file annotation, and it includes the information of coordinates (Xmin, Ymin, Xmax, Ymax) of mushroom. According to this information, the height and width of bounding box can be calculated. Fig. 4 shows the Pascal VOC annotation format.

**Fig. 5:**
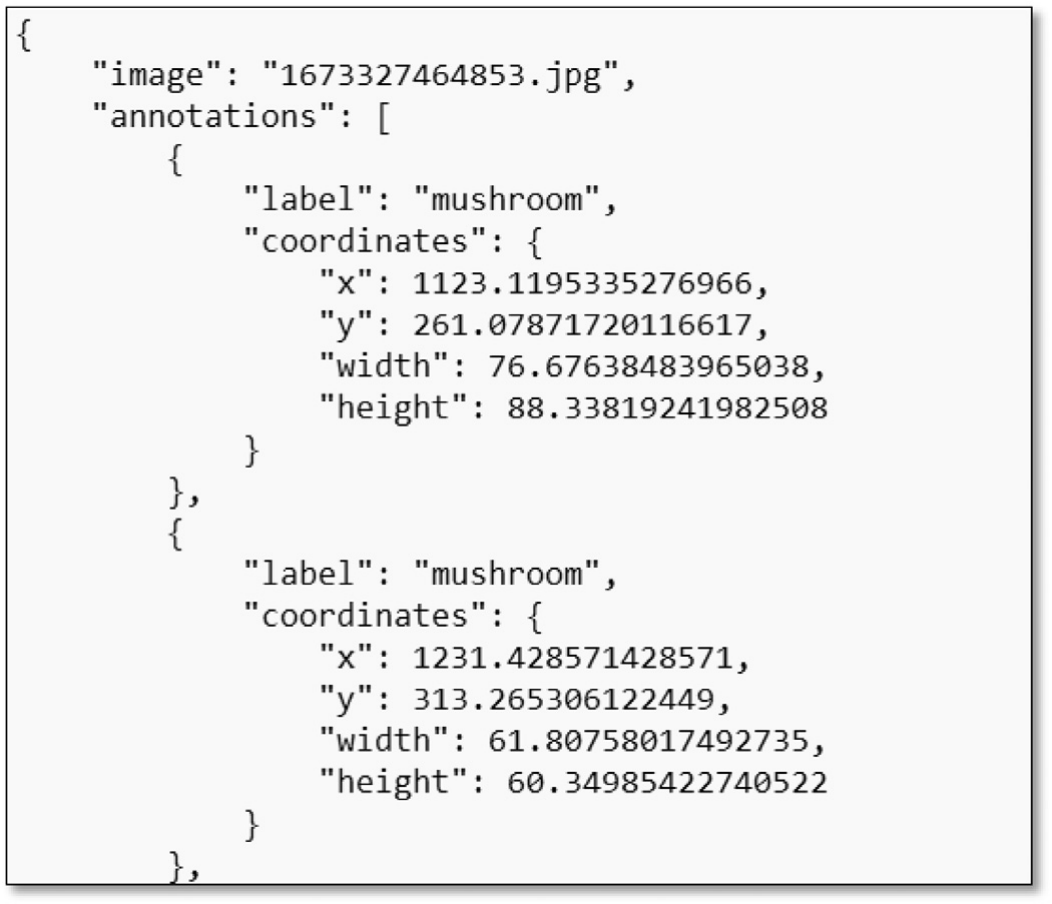
COCO annotation format.

The COCO (Common Objects in Context) format [5] is widely adopted as the standard data format for training and inference in object detection tasks, and it is required that all data related to object detection tasks conform to the COCO format. The COCO format is a JSON (JavaScript Object Notation) structure that governs how labels and metadata are formatted for a dataset, and it is one of the most popular datasets for object detection. The JSON file includes the x and y coordinates of mushroom and also, the height and width of them, Fig. 5.

**Fig. 6:**
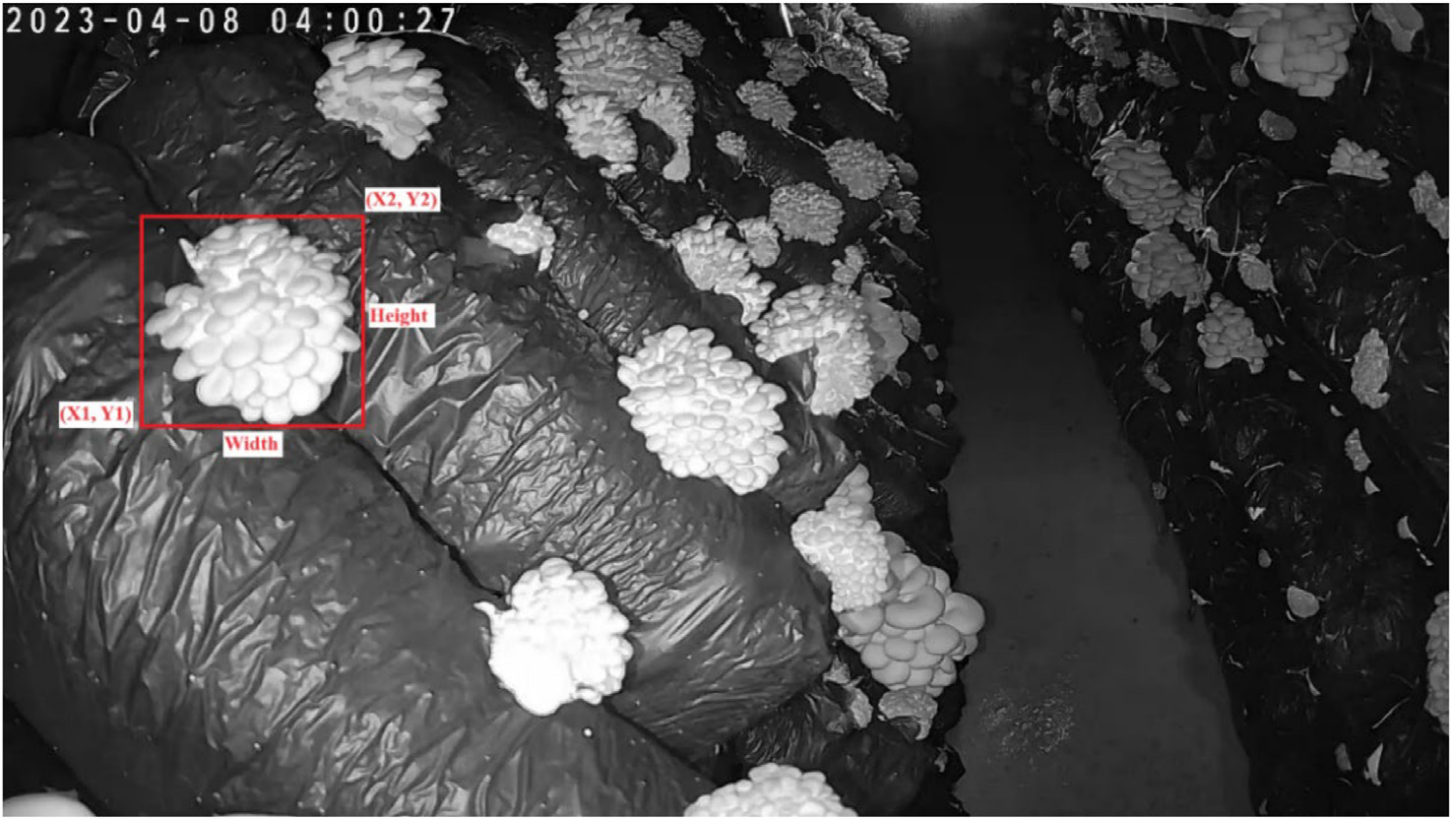
YOLO Darknet annotation format representation.

The YOLO Darknet [6] contains one text (TXT) file per image, which includes the annotations and a numeric representation of the label, as well as a label map that maps the numeric IDs to human-readable strings. The annotations are normalized to lie within the range [0,1], making them easier to work with even after scaling or stretching images. Its popularity has grown due to its alignment with the Darknet framework implementations of the various YOLO models. Fig. 6 shows the YOLO Darknet annotation format on an example image.

The collected and processed data was organized into four separate folders: raw images, mushroom labelled images, maturity (mature and immature) labelled mushroom images, sensor data of the farm. Table 1 shows a brief description of the dataset folders and files.

I. **Raw Images**
  This folder contains 555 images in JPG format. These images were selected from 1200 images taken by the two cameras, when the image contains mushrooms in it.
II. **Mushroom Labelled Images and Annotation Files**
  This folder contains a sub-folder with 7963 images in JPG format, in addition to 3 subfolders for JSON, XML, and TXT formatted annotation files of all raw images. The raw images were cropped to get mushroom images only and labelled as “mushroom” using LabelMe software, Fig. 5.
III. **Maturity Labelled Images and Annotation Files**
  This folder contains 5 sub-folders; the first one contains 5124 images of mushrooms labelled as “immature”, and the second one contains 3158 images of mushroom labelled as “mature”. Additionally, there are 3 sub-folders for JSON, XML, and TXT formatted two-class annotation files of all raw images.
IV. **Sensor Data**
  This folder contains two sub-folders of environmental data, obtained from different sensors during two cycles. The Cycle I folder contains 4 Excel files and 388 .csv files representing time-stamped sensor data. Cycle II contains 11 Excel files to represent the sensor values by date and time. These environmental data include temperature (DHT Temp, SHT Temp), relative humidity (DHT Hum, SHT Hum), and air quality (SGP ECO2, SGP TVOC, CSS ECO2, CSS TVOC) in the mushroom greenhouse, in addition to temperature (DS Temp) and moisture (Cap Moist, Res Moist) inside some mushroom composite bags.

**Table 1:** Brief description of the dataset folders/files.

No.	Name	Type/Format	Description	Size
1	Full Dataset	Root folder	Conveniently packaged for download	476 MB
2	Raw Images	Compressed (.rar) folder: 555 JPG images	Original camera images with a lot of mushrooms	268 MB
3	Mushroom Labelled Images and Annotation Files	Compressed (.rar) folder: Mushroom images sub-folder with 7963 JPG images- Three sub-folders: COCO, PASCAL VOC, and YOLO for one-class annotation files of all raw images	Using LabelMe, raw images were cropped to get mushroom images and labelled as “mushroom” in annotation files of different (JSON, XML and TXT) formats	80 MB
4	Maturity Labelled Images and Annotation Files	Compressed (.rar) folder: Two mature and immature sub-folders with 8282 JPG images- Three sub-folders: COCO, PASCAL VOC, and YOLO for two-class annotation files for all raw images	Using LabelMe, the images initially taken from raw images were classified into two classes and labelled as “mature” and “immature” in annotation files of different (JSON, XML and TXT) formats	80 MB
5	Sensor Data	Compressed (.rar) folder: Sub-folders (Cycle I & Cycle II): −15 Excel .xlsx files- Sub-folder with a .txt notes file and 97 .zip files for daily data.	Sensor environmental values of the farm: Excel .xlsx files for ThingSpeak data- Compressed .zip files for Arduino IoT Cloud daily data with 388 .csv files and 97 .txt readme files	53 MB

## 4 Experimental Design, Materials and Methods

**Fig. 7:**
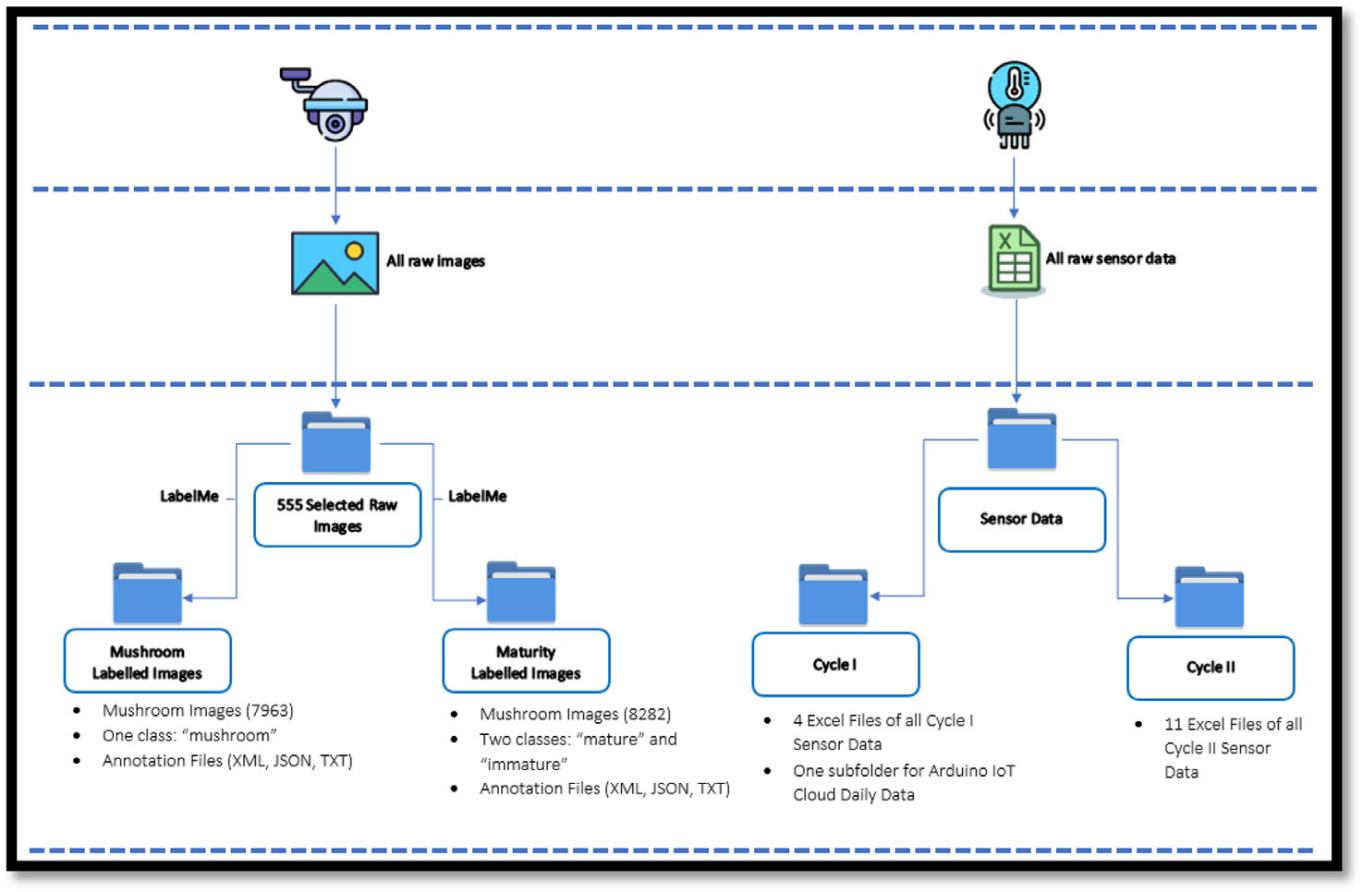
Construction and annotation of dataset.

Fig. 7 shows the structure and construction methodology of our dataset of annotated mushroom images with their environmental context.

The original raw images were taken using two IP cameras with night and day vision in an oyster mushroom cultivation farm in Mersin, Turkey, on the northern Mediterranean coast. There were at least 4–5 images per day, in addition to associated environmental data obtained using an IoT-based system [2] in the farm for two mushroom cultivation cycles from late December 2022 until late April 2023.

In this study, mushroom clusters in the raw images, Fig. 1, were manually selected and turned into separate images, such as those shown in Fig. 2. At the same time, annotation files with three different (XML, JSON, and TXT) formats, which are widely adopted in object detectors, were created for all raw images. With YOLO Darknet, COCO and PASCAL VOC formats, researchers will be able to use this dataset for different machine learning and deep learning studies.

In a similar dataset of oyster mushroom images published by Sujatanagarjuna et al. [7], a total of 34,400 raw, unannotated mushroom images were collected in an amateur cultivation environment using plastic buckets. However, only 3300 mushrooms were annotated (2759 for training and 541 for testing) using the COCO (JSON) annotation format. In contrast, this dataset contains 7963 annotated mushrooms and 8282 maturity-labelled mushrooms using three different annotation formats. Additionally, and more importantly, the associated environmental data, such as temperature, relative humidity, air quality inside the greenhouse, as well as temperature and moisture inside mushroom composite bags were recorded at one-minute intervals using various sensors to assess their impact on mushroom growth using machine learning techniques.

This dataset serves as an invaluable resource for researchers aiming to develop high-performance machine-learning models for intelligent oyster mushroom cultivation. Beyond its immediate application, the dataset holds promising aspects for exploration in diverse fields, including computer vision, precision agriculture, robotics, and broader fungal studies. Overall, this initiative contributes significantly to advancing mushroom-related research and technology.

## Limitations

Environmental data were collected over two cultivation cycles. In the first cycle, the temperature and relative humidity inside the mushroom greenhouse were measured using cost-efficient DHT21 sensors. In the second cycle, the more reliable SHT20 sensor was added, along with air quality sensors, for more trustworthy and comprehensive environmental data. More technical details are available in [2].

## Ethics Statement

The expected standards of ethical behaviour in scientific publishing were generally adhered to by the authors throughout the construction of the article. The work did not entail the use or involvement of human subjects or animals, thereby aligning with ethical guidelines for research conduct.

## CRediT Author Statement

**Sonay Duman:** Writing – original draft, Software, Data curation, Investigation. **Abdullah Elewi:** Writing – review & editing, Validation, Resources, Project administration, Methodology, Conceptualization. **Abdulsalam Hajhamed:** Validation, Resources, Conceptualization, Funding aquisition. **Rasheed Khankan:** Software, Data curation, Investigation. **Amina Souag:** Writing – review & editing, Validation, Methodology, Conceptualization, Supervision. **Asma Ahmed:** Writing – review & editing, Conceptualization, Supervision.

## Data Availability

Mendeley DataAnnotated oyster mushroom images (Original data).
